# Clinical practice guidelines for safe performance of endoscopic ultrasound/ultrasonography‐guided biliary drainage: 2018

**DOI:** 10.1002/jhbp.631

**Published:** 2019-06-28

**Authors:** Hiroyuki Isayama, Yousuke Nakai, Takao Itoi, Ichiro Yasuda, Hiroshi Kawakami, Shomei Ryozawa, Masayuki Kitano, Atsushi Irisawa, Akio Katanuma, Kazuo Hara, Takuji Iwashita, Naotaka Fujita, Kenji Yamao, Masahiro Yoshida, Kazuo Inui, Masanori Sugiyama, Keiichi Kubota, Tadahiro Takada

**Affiliations:** ^1^ Department of Gastroenterology Graduate School of Medicine Juntendo University Tokyo Japan; ^2^ Department of Gastroenterology Graduate School of Medicine The University of Tokyo Tokyo Japan; ^3^ Department of Gastroenterology and Hepatology Tokyo Medical University Tokyo Japan; ^4^ Third Department of Internal Medicine University of Toyama Toyama Japan; ^5^ Division of Gastroenterology and Hepatology Department of Internal Medicine Faculty of Medicine University of Miyazaki Miyazaki Japan; ^6^ Department of Gastroenterology Saitama Medical University International Medical Center Saitama Japan; ^7^ Second Department of Internal Medicine Wakayama Medical University Wakayama Japan; ^8^ Department of Gastroenterology Dokkyo Medical University Tochigi Japan; ^9^ Center for Gastroenterology Teine‐Keijinkai Hospital Sapporo Japan; ^10^ Department of Gastroenterology Aichi Cancer Center Hospital Nagoya Japan; ^11^ First Department of Internal Medicine Gifu University Hospital Gifu Japan; ^12^ Miyagi Health Check‐up Plaza Sendai Japan; ^13^ Department of Gastroenterology Narita Memorial Hospital Nagoya Japan; ^14^ Department of Hepato‐Biliary‐Pancreatic and Gastrointestinal Surgery School of Medicine International University of Health and Welfare Ichikawa Japan; ^15^ Department of Gastroenterology Fujita Health University Bantane Hospital Nagoya Japan

**Keywords:** Biliary stricture, Endoscopic ultrasonography, EUS‐guided biliary drainage, Interventional EUS

## Abstract

Endoscopic ultrasound/ultrasonography‐guided biliary drainage (EUS‐BD) is a relatively new modality for biliary drainage after failed or difficult transpapillary biliary cannulation. Despite its clinical utility, EUS‐BD can be complicated by severe adverse events such as bleeding, perforation, and peritonitis. The aim of this paper is to provide practice guidelines for safe performance of EUS‐BD as well as safe introduction of the procedure to non‐expert centers. The guidelines comprised patient–intervention–comparison–outcome‐formatted clinical questions (CQs) and questions (Qs), which are background statements to facilitate understanding of the CQs. A literature search was performed using the PubMed and Cochrane Library databases. Statement, evidence level, and strength of recommendation were created according to the GRADE system. Four committees were organized: guideline creation, expert panelist, evaluation, and external evaluation committees. We developed 13 CQs (methods, device selection, supportive treatment, management of adverse events, education and ethics) and six Qs (definition, indication, outcomes and adverse events) with statements, evidence levels, and strengths of recommendation. The guidelines explain the technical aspects, management of adverse events, and ethics of EUS‐BD and its introduction to non‐expert institutions.

## Graphical abstract

***Highlight***

Isayama and colleagues present the first clinical practice guidelines for endoscopic ultrasound‐guided biliary drainage (EUS‐BD), including indications, clinical results, procedures, devices and education. Their primary purpose is to improve the outcomes of EUS‐BD for biliary tract diseases by facilitating the safe introduction and performance of the procedure at non‐expert centers.

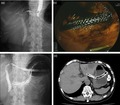

## Introduction

Endoscopic biliary drainage for obstructive jaundice and acute cholangitis includes biliary stenting and endoscopic nasobiliary drainage by endoscopic retrograde cholangiopancreatography (ERCP). However, these techniques cannot be applied in some patients with difficult or impossible biliary cannulation, gastric outlet obstruction, or a surgically altered anatomy. Since Wiersema et al. 1 first reported endoscopic ultrasound (EUS)‐guided cholangiopancreatography in 1996, several modifications of EUS‐guided biliary drainage (EUS‐BD), such as EUS‐guided bilioduodenal anastomosis 2, which creates a fistula between the common bile duct and duodenum, have been made, and the use of this technique in clinical practice is increasing rapidly. However, the techniques and devices require further improvement for safe and widespread use of EUS‐BD, for which the current guidelines provide critical information.

The purpose of these guidelines is to provide information on the safety of EUS‐BD and on the methods for its introduction to more institutions, with the aim of improving clinical practice and promoting safe performance of the technique. The steering committee expects that the guidelines will promote widespread and safe performance of EUS‐BD in a large number of institutions. These guidelines are targeted to patients and their families, physicians, and other medical staff, to provide information on the current status of EUS‐BD. The guidelines also aim to improve the outcomes of EUS‐BD for biliary tract diseases, focusing on biliary obstruction, including obstructive jaundice and acute cholangitis. Since EUS‐BD is still in the introduction phase and high‐quality evidence is lacking, our guidelines need to be updated as novel dedicated devices are developed and more evidence is accumulated in the future.

## Methods

### Committee organization and approval process of consensus among committee members

A guideline creation committee, expert panelist committee, evaluation committee, and external evaluation committee were organized (Appendix 2). Because the guidelines focus on the technical aspects of EUS‐BD, members of the guideline creation and expert panelist committees were selected from among gastroenterologists with expertise in endoscopy and interventional radiology (IVR). Two external evaluation committee members who are experts in the guideline creation process were invited to evaluate the guidelines and provide recommendations for their improvement. The first step in creating the guidelines was to identify clinically important issues and generate patient–intervention–comparison–outcome‐formatted clinical questions (CQs) and evaluate the evidence in a systematic review. The next step was to evaluate the clinical evidence, risk–benefit balance, wishes of the patients, and medical costs. The third step was to generate the statements. However, few studies on EUS‐BD have provided high‐quality evidence. Therefore, the statements, recommendation levels, and evidence levels were determined based on the GRADE system, which is described in detail later, and were decided by an expert consensus survey. Voting was performed via the internet to avoid the influence of other committee members’ opinions. The statements in each CQ were assigned a score of 9 or lower, and the voting was repeated until the mean score reached ≥7.

### Process of guideline creation

#### Creation of CQs and Qs

Draft patient–intervention–comparison–outcome‐formatted CQs for important clinical subjects and Qs for background information to understand the CQs were generated. Thirteen CQs and six Qs were generated by the guideline committee, and one member was assigned to each question.

#### Evidence collection

The committee member responsible for each CQ performed a systematic literature search in PubMed and the Cochrane Library and produced a reference list based on the content and design of each identified study. The literature search was limited to the period from January 1990 to May 2018.

#### Method of evidence assessment and systematic review

The evidence was summarized based on the systematic review of the literature related to EUS‐BD. The evidence levels and recommendation levels determined using the GRADE system are listed in Tables 1 and 2.

**Table 1:** Methods of decision of evidence levels according to the Grade system

Initial quality of evidence	Study design	Lower if	Higher if
High	RCT, systematic review, meta‐analysis	Study limitations: 1 Serious 2 Very serious Inconsistency: 1 Serious 2 Very serious Indirectness: 1 Serious 2 Very serious Impression: 1 Serious 2 Very serious Publication bias: 1 Likely 2 Very likely	Magnitude of effect: 2 Very strong 1 Strong Dose‐response gradient 1 All plausible confounders would have reduced the effect 1
Moderate			
Low	Observational study (cohort study, case control study)		
Very low	Any other evidence (case series, case study)		

*Level A* high, *Level B* moderate, *Level C* low, *Level D* very low

Overall quality of evidence across studies for the outcome

**Table 2:** GRADE system (grade of recommendation)

1. How to judge a Grade of recommendation	
Total judgment with evidence, harm and benefit	
Level of evidence	A, B, C, D
Patient's preference	Yes, No
Harm and benefit	Yes, No
Cost effectiveness	Yes, No
2. How to show a Grade of recommendation: 2 steps	
Recommendation 1: Strong recommendation (do it, don't do it)	
Recommendation 2: Weak recommendation (probably do it, probably don't do it)	

#### Decision of statements and creation of draft guidelines

First, a draft statement of each CQ and Q was created based on the risk–benefit balance, wishes of the patients, and medical costs; the evidence level was appended as a note (first draft). After discussion among the guideline creation committee members, revised statements (second draft) were preliminarily voted on by the expert committee via the internet as a consensus survey. Agreement of the statements was defined as a mean score for all CQs of ≥7, and expository text was created for each CQ. Next, three face‐to‐face meetings were held to revise each statement and its commentary text (third draft). The expert panelist committee voted via the internet, and the third draft statements and commentary text approved by consensus were added. The third draft was further revised according to the opinions of the expert panelist committee; in this way, the draft statements, evidence levels, and commentary text were created.

#### Creation of the final draft and the finalization process

The draft guidelines were proposed to the public on 29 September 2018 at the 52nd Japan Biliary Association Annual Scientific Meeting. The draft was revised based on the collected comments and opinions, and the revised draft was further reviewed and revised by the evaluation committee. The final draft was posted on the website of the Japan Biliary Association from 24 August to 30 September 2017. The public comments received were incorporated into the final draft, which was further revised and/or corrected and modified with the approval of the guideline creation committee. The guidelines were finalized according to the evaluation by and advice of the members of the external evaluation committee.

### The GRADE system

In the guidelines, the evidence grade was defined according to the GRADE system, as follows: A, high (strong evidence); B, moderate (moderate evidence); C, low (low evidence); and D, very low (weak evidence). The recommendation grades are 1 (strong recommendation) and 2 (weak recommendation). Table 1 lists the quality‐of‐evidence and grade levels of the GRADE system 3, 4, 5, and Table 2 shows the GRADE system recommendations 3, 4, 5.

## Questions and clinical questions of the guidelines

### **Q1. What is endoscopic ultrasound‐guided biliary drainage (EUS‐BD)?**

**EUS‐BD is defined as transmural biliary access under endoscopic ultrasound guidance with subsequent biliary drainage.**

**Mean voting score (range): 7.7 points (1–9)**

EUS‐BD for obstructive jaundice is an endoscopic transmural biliary drainage approach to the biliary tract under EUS guidance. An ERCP approach is widely used for the diagnosis and treatment of pancreatobiliary diseases. For obstructive jaundice, transpapillary endoscopic biliary drainage (EBD) is the first choice. For patients in whom EBD is difficult, percutaneous transhepatic biliary drainage (PTBD) or hepaticojejunostomy is performed. However, these procedures are highly invasive, have risks of complications, and affect the quality of life of the patient. Thus, EUS‐BD was developed as an alternative drainage modality.

**Figure 1:**
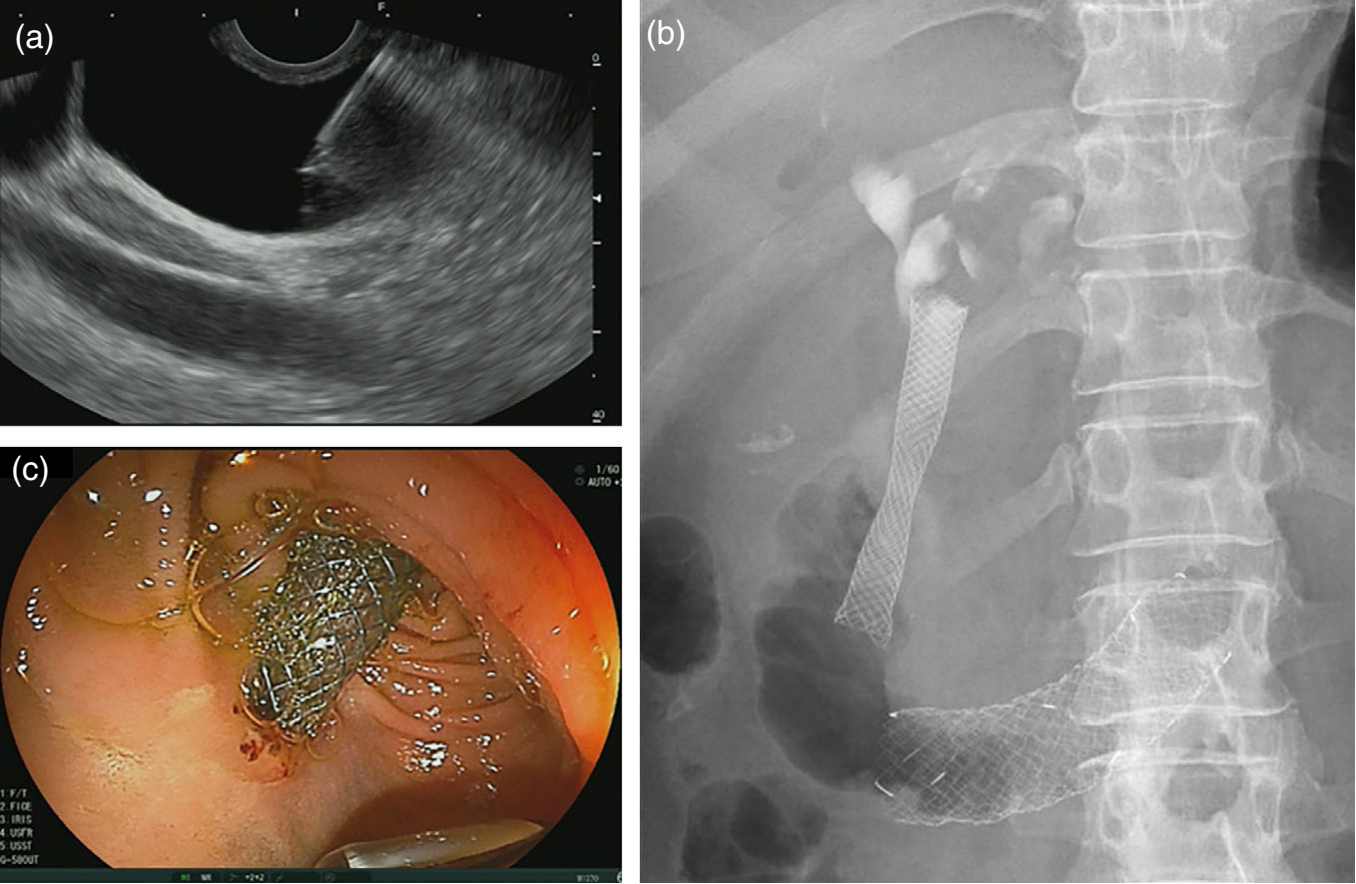
EUS‐guided choledochoduodenostomy (EUS‐CDS). (**a**) Puncture of the common bile duct under EUS‐guidance. (**b**) Fluoroscopy after EUS‐CDS stent placement. (**c**) Endoscopic view after EUS‐CDS stent placement

**Figure 2:**
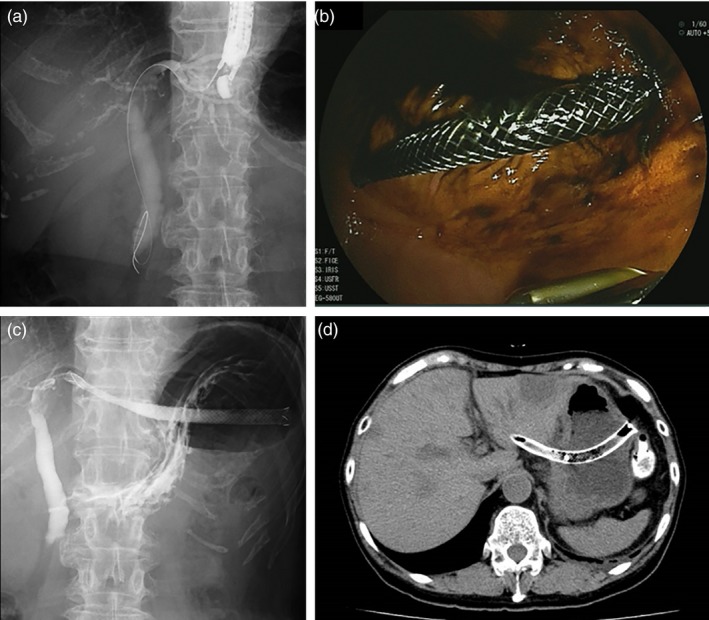
EUS‐guided hepaticogastrostomy (EUS‐HGS). (**a**) Fluoroscopy of EUS‐guided puncture of the left intrahepatic bile duct. (**b**) Endoscopic view after EUS‐HGS. (**c**) Fluoroscopy after EUS‐HGS. (**d**) CT image of the EUS‐HGS stent

EUS‐BD is either a transmural biliary drainage that creates a fistula between the gastrointestinal tract and the biliary tract (including EUS‐guided choledochoduodenostomy [CDS, Fig. 1] and EUS‐guided hepaticogastrostomy [HGS, Fig. 2]) or a transpapillary or antegrade approach after EUS‐guided puncture of the bile duct (including EUS‐guided rendezvous [EUS‐RV] and EUS‐guided antegrade stenting [EUS‐AGS]). The procedure is selected according to the patient's background, treatment indications, and difficulty of endoscopic retrograde cholangiography (ERC). EUS‐guided gallbladder drainage can be used if bile duct puncture is difficult, and biliary drainage is performed via the gallbladder. This procedure is also carried out for acute cholecystitis.

Wiersema et al. 1 first reported EUS‐guided cholangiography in 1996. In that study, a 22‐ or 23‐gauge (G) needle for fine‐needle aspiration (FNA) was used for cholangiography in 10 cases and for pancreatography in one case; the procedure was successful in eight cases. In 2001, Giovaninni et al. 2 reported on EUS‐guided bilioduodenal anastomosis, namely EUS‐CDS, in which a fistula was created between the common bile duct and the duodenum for biliary drainage. In 2003, Burmester et al. 6 reported four cases with EUS‐guided cholangio‐drainage and one with intrahepatic bile duct puncture, namely EUS‐HGS, via the transgastric approach. Since the terms “EUS‐CDS” and “EUS‐HGS” are widely used globally, the current guidelines also used these terms. However, in EUS‐CDS, the bile duct was punctured not only at “choledocus (the common bile duct)” but also the common hepatic duct, and the term “EUS‐CDS” does not always represent the accurate anatomy. Although there are some opinions that the term should be either “intrahepatic” or “extrahepatic” to accurately describe the puncture site, there is no consensus on this and the terms EUS‐CDS and HGS are widely used in clinical practice.

In addition to transmural biliary drainage, Mallery et al. 7 in 2004 reported on biliary drainage using EUS‐RV, in which needle puncture into the bile duct is performed under EUS guidance followed by selective biliary cannulation using a guidewire advanced into the duodenum. Fujita et al. 8 reported a two‐stage stent placement procedure in which a guidewire was inserted into the bile duct through a temporary fistula created by EUS‐HGS to the duodenum in an antegrade manner, followed by placement of a metal stent in the bile duct, similar to EUS‐AGS. In 2010, Nguyen‐Tang et al. 9 reported five cases of EUS‐guided antegrade stent placement. In addition to the procedures described above, EUS‐guided hepaticojejunostomy 6 from the jejunum to the intrahepatic bile duct in cases with surgically altered anatomy and EUS‐hepaticoduodenostomy 10 from the duodenum to the intrahepatic bile duct have also been reported.

### **Q2. What are the indications for EUS‐BD?**

**EUS‐BD is used in patients with failed or difficult ERC who require biliary drainage.**

**Mean voting score (range): 8.3 points (7–9)**

EUS‐BD can be applied in patients with failed or difficult ERC, and unresectable malignant biliary obstruction can be a good indication 11, 12, 13, 14, 15, 16, 17, 18. The causes of failed or difficult ERC include failed biliary drainage for various anatomical reasons, difficult endoscope advancement due to duodenal stenosis by tumor invasion, an indwelling duodenal stent for gastric outlet obstruction, or a surgically altered anatomy. The success rate of EBD is 84.0–95.6% 19, 20, and additional procedures, including precut sphincterotomy, can increase the success rate of biliary cannulation to 80.2–100% 21. The rate of patients with failed or difficult ERC who required EUS‐BD is reportedly 0.6% (3/524 cases) at high‐volume tertiary‐care centers 22. Thus, few patients require EUS‐BD as a salvage procedure.

Although EUS‐BD has been used as the first‐line drainage method 23, 24, consensus on its use in cases in which ERC can be performed has not been established. Kawakubo et al. 25 retrospectively compared the clinical effectiveness and complication rates of EUS‐CDS (*n* = 26) and EBD (*n* = 56) in patients with unresectable malignant distal biliary obstruction. While the clinical effectiveness (96.2% vs. 98.2%; *P* = 0.54) and complication (26.9% vs. 35.7%; *P* = 0.46) rates were comparable, EUS‐CDS had a shorter procedure time (19.7 vs. 30.2 min; *P* < 0.01), and none of the patients developed pancreatitis. Hamada et al. 26 compared EUS‐BD (*n* = 7) and EBD (*n* = 13) in patients with duodenal stent placement. The stent failure and complication rates were similar, but the stent‐patency rates at 1 month (100% vs. 71%) and 3 months (83% vs. 29%) were higher for EUS‐BD. More recently, three randomized controlled trials 27, 28, 29 comparing EUS‐BD and ERCP‐biliary drainage were published (Table 3). Although all studies demonstrated comparable clinical outcomes, the studies were underpowered by a small sample size and a large scale prospective study is warranted.

**Table 3:** Summary of randomized controlled trials of EUS‐BD versus ERCP or PTBD

Author	Intervention	*n*	Technical success (%)	Clinical success (%)	Adverse events (%)	Stent patency
Paik 27	EUS‐BD	61	93.8	90.0	6.3[Link]	208 days
	ERCP	61	90.2	94.5	19.7	165 days
Park 28	EUS‐BD	14	92.8	100	0	379 days
	ERCP	14	100	92.8	0	403 days
Bang 29	EUS‐BD	33	90.9	97.0	21.2	182 days
	ERCP	34	94.1	91.2	14.7	170 days
Artifon 36	EUS‐BD	13	100	100	15.3	–
	PTBD	12	100	100	25	–
Lee 35	EUS‐BD	34	94.1	87.5	8.8[Link]	–
	PTBD	32	96.9	87.1	31.2	–

*ERCP* endoscopic retrograde cholangiopancreatography, *EUS‐BD* endoscopic ultrasound‐guided biliary drainage, *PTBD* percutaneous transhepatic biliary drainage

*P* < 0.05

EUS‐BD can be the optimal treatment modality for patients in which EBD is difficult to perform due to a surgically altered anatomy. Siripun et al. 30 performed a systematic review of EUS‐BD and found that the success, clinical effectiveness, and complication rates were 89.2%, 91.7%, and 17.5%, respectively, similar to those of EUS‐BD. In an international, retrospective study comparing EUS‐BD and enteroscopy‐assisted ERCP in 98 surgically altered anatomy cases 31, EUS‐BD was associated with high technical success rate (odds ratio [OR] 12.48, *P* = 0.001) but the adverse event rate was higher in EUS‐BD (20% vs. 4%; *P* = 0.01). However, technical success rates of EUS‐BD and enteroscopy‐assisted ERCP might differ by local expertise and a further prospective study is warranted. However, there are no prospective studies comparing EUS‐BD and enteroscopy‐assisted ERCP; therefore, further studies are necessary.

Ogura et al. 32 reported successful re‐intervention using EUS‐BD in patients with malignant hilar biliary obstruction and stent failure; the success rate of re‐intervention by ERC was 62% (16/26), and EUS‐BD was successful in all 10 cases of ERC failure. These cases may have relative indication for EUS‐BD because the procedure has not yet been established, and such patients frequently have a complex pathology. Thus, EUS‐BD should be performed by experts only.

There are few studies of EUS‐BD for resectable malignant biliary obstruction; therefore, the evidence level is low. Fujita et al. 33 performed EUS‐CDS followed by surgical resection and pathological examination; none of the patients displayed severe inflammation or bile leakage. However, performing EUS‐BD in resectable cases is not recommended.

The indications for EUS‐BD for benign biliary stricture include a surgically altered anatomy (for which conventional ERC is unsuitable), bile duct stones, biliary stricture with chronic pancreatitis, post‐liver transplantation biliary stricture, or IgG4‐associated cholangitis 15, 16, 18, 34. However, there are few case reports, and the long‐term outcomes are unclear. Thus, the indications for EUS‐BD should be determined.

A randomized controlled trial of PTBD (*n* = 32) versus EUS‐BD (*n* = 34) showed similar technical success (96.9% vs. 94.1%) and clinical success (87.1% vs. 87.5%) rates, but PTBD had a significantly higher adverse event rate (31.2% vs. 8.8%; *P* = 0.022) and required more re‐interventions (0.93 vs. 0.34; *P* = 0.020) 35. A prospective study of 25 cases reported similar treatment efficacy and safety rates between PTBD and EUS‐BD 36. Another retrospective study of 41 cases concluded that EUS‐BD showed better outcomes than those of PTBD 37. A randomized controlled study comparing EUS‐CDS with surgical bypass in 29 cases reported similar technical success, clinical success, and survival rates and quality of life 38. Sharaiha et al. 39 performed a meta‐analysis of 483 cases and found that whereas the technical success rates were similar (OR 1.78; 95% CI 0.69–4.59), EUS‐BD demonstrated better clinical success (OR 0.45; 95% CI 0.23–0.89) and adverse event (OR 0.23; 95% CI 0.12–0.47) rates compared with PTBD. Although EUS‐BD has equivalent or better outcomes than percutaneous or surgical approaches, a larger‐scale prospective comparative study is necessary to acquire sufficient evidence.

Although the indications for EUS‐BD will likely change as techniques advance, it is important to determine the indications by considering the condition of the patients and the skill and experience of the endoscopist. In addition, EUS‐BD for non‐established indications should be evaluated in clinical trials, not in clinical practice.

### **Q3. What are the contraindications for EUS‐BD?**

**Contraindications for EUS‐BD include conditions that prevent visualization of the bile duct by EUS, intervening vessels and/or other organs, bleeding tendency, and/or excess ascites.**

**Mean voting score (range): 8.4 (7–9)**

Although there is no clear evidence of contraindications for EUS‐BD, they are similar to those of EUS‐FNA and PTBD. In patients with excess ascites, a mature fistula cannot be created after EUS‐BD, and there is a risk of peritonitis due to leakage of bile and intestinal contents. Although there is no clear definition of the amount of ascites, EUS‐BD should not be performed in cases with excess ascites or ascites present in the puncture route. In addition, the indications for EUS‐BD should be carefully assessed even in cases with a small amount of ascites or without ascites in the puncture route. Evaluation of the general condition of the patient during EUS‐BD is important because of the potential need for surgical intervention due to unsuccessful EUS‐BD or adverse events. EUS‐BD should not be used in patients with poor conditions without careful consideration and is contraindicated for patients who have or are suspected to have tumor invasion in the puncture site.

There is no clear evidence of the utility and safety of EUS‐BD in patients on antithrombotic agents. In the 2012 Guidelines for Gastroenterological Endoscopy in Patients Undergoing Antithrombotic Treatment of the Japan Gastroenterological Endoscopy Society 40, EUS‐FNA is classified as having a high risk of bleeding. Thus, EUS‐BD should also be considered to have a high risk of bleeding. Among patients who underwent EUS‐FNA, the incidence rates of bleeding in those on aspirin/non‐steroid anti‐inflammatory drugs, low‐molecular‐weight heparin, and controls were 0% (0/26), 33.3% (2/6), and 3.7% (7/190), respectively. Therefore, the bleeding rate was not significantly higher in the aspirin/non‐steroid anti‐inflammatory drug group 41. However, in a large‐scale analysis of PTBD using the Japanese DPC database of 34,606 cases 42, the rate of severe hemorrhage was significantly higher in patients on compared with those not on anti‐thrombotic agents (OR 1.87; 95% CI 1.14–3.05; *P* = 0.013).

### **Q4. What are the short‐term outcomes of EUS‐BD?**

**The technical and clinical success rates of EUS‐BD in experienced centers are ≥90%.**

**Mean voting score (range): 8.1 (5–9)**

The short‐term outcomes of EUS‐BD include the rates of technical and clinical success. Adverse events are described in Q6. In general, technical success is defined as successful biliary drainage as planned and clinical success as relief of jaundice and cholestasis.

The success rates of EBD, which is the first‐choice procedure for biliary drainage, and PTBD in patients in whom ERC would be problematic are high. The technical and clinical success rates of CDS and HGS are also ≥90% 13, 34, 43. Two previous systematic reviews 44, 45 reported technical success rates of 94.71% and 90% and a clinical success rate of 91.66%. EUS‐HGS and EUS‐CDS have similarly high technical and clinical success rates. In a multicenter retrospective study of 64 Japanese patients who underwent EUS‐BD (44 CDS, 20 HGS) 34, the technical success rate was 95% for both EUS‐CDS and EUS‐HGS. These results are similar to those among non‐Japanese patients. In a recent systematic review 46, technical success rates of CDS and HGS were 94.1% and 93.7%, respectively. Clinical success rates were also comparable: 88.5% in CDS and 84.5% in HGS (Table 4).

**Table 4:** Summary of technical and clinical success rates of EUS‐BD

	Technical success (%)	Clinical success (%)
EUS‐CDS 46	94.1	88.5
EUS‐HGS 46	93.7	84.5
EUS‐RV 47	82	–
EUS‐AG stenting 9, 15, 43, 49, 50, 51, 52	83	–

*AG* antegrade, *CDS* choledochoduodenostomy, *EUS* endoscopic ultrasound, *HGS* hepaticogastrostomy, *RV* rendezvous

In EUS‐BD other than transmural drainage, the success rate of EUS‐RV, which converts to a transpapillary approach after EUS‐guided biliary access, is 82% 47. The success rate of the intrahepatic bile duct approach is 76%, which is lower than that of the extrahepatic bile duct approach (85%). Prior studies involved patients with not only malignant biliary obstruction but also those with bile duct stones, and the lower success rate was due to guidewire manipulation to pass the biliary stricture or ampulla 48. In studies of EUS‐AGS 9, 15, 43, 49, 50, 51, 52, the success rate was 83%, similar to that of EUS‐RV, but conversion to transmural biliary drainage was reported in cases in which the guidewire could not be manipulated across the biliary stricture 51. However, those results were reported by experienced endoscopists, and the success rate was lower in a less‐experienced center 16, suggesting publication bias. Regarding the learning curve of EUS‐BD, a mortality rate of 10% during the initial phase was reported 18. A single center study in Thailand of 31 cases over 5 years reported a technical failure rate of 38% during the initial 3 years, compared with 11% during the last 2 years. The adverse event rate decreased from 54% to 22% 53, suggesting a steep learning curve for EUS‐BD, which should be taken into consideration when introducing EUS‐BD to new centers.

Due to the difficulty of biliary cannulation in EBD, comparative studies of the short‐term outcomes of EUS‐BD and EBD for malignant distal biliary obstruction have been performed. A multicenter retrospective study 43 showed similar technical success and adverse event rates between 104 EUS‐BD cases (68 EUS‐CDS and 36 EUS‐AGS cases) and 104 EBD cases (93.26% vs. 94.23% success rate and 8.65% vs. 8.65% adverse event rate) and a significantly higher success rate for EUS‐BD in cases with duodenal stenosis compared with EBD. A Japanese single‐center retrospective study 25 compared EUS‐CDS (*n* = 26) and EBD (*n* = 56) performed for primary drainage of malignant distal biliary obstruction. Whereas the clinical success rates were similar (96.2% vs. 98.2%), the procedure time of EUS‐CDS (19.7 min) was shorter than that of trans‐papillary drainage (30.2 min). Both studies reported no post‐procedural pancreatitis resulting from EUS‐BD. Comparative data of EUS‐BD and ERCP are shown in Table 3. The type of adverse events of EUS‐BD and ERCP differ but the adverse event rates were comparable or even lower in EUS‐BD in randomized controlled trials 27, 28, 29. While post‐ERCP pancreatitis is a major adverse event after ERCP, bile leak is a potential severe adverse event after EUS‐BD.

Therefore, EUS‐BD can be a first‐line option, irrespective of the difficulty of ERC. Prospective studies in Japanese expert centers have reported favorable short‐term outcomes 23, 24. However, because the evidence supporting the use of EUS‐BD as the primary drainage procedure is insufficient, further clinical studies should be conducted in patients other than those in whom ERC is technically or clinically difficult or impossible.

### **Q5. What are the long‐term outcomes of EUS‐BD?**

**The few studies on the mid‐ and long‐term outcomes of EUS‐BD reported a stent patency of 3–6 months.**

**Mean voting score (range): 8.1 (5–9)**

Because EUS‐BD is a relatively new procedure, most previous studies have focused on the technical success and procedure‐related adverse event rates, whereas few have evaluated the long‐term outcomes. Prior studies using various drainage routes and stent types found stent occlusion rates of 16% (19% for CDS, 13% for HGS) and stent patency of 3–6 months (Table 5) 11, 18, 23, 24, 26, 34, 54, 55, 56, 57, 58, 59, 60, 61, 62, 63, 64, 65, 66, 67. In EUS‐RV or EUS‐AGS, the biliary drainage route is similar to that of transpapillary drainage, and thus the mid‐ and long‐term outcomes are expected to be similar to transpapillary drainage.

**Table 5:** Long term outcomes of EUS‐BD

	Occlusion rate of stent	Patency period
Total	16% (95% CI 13–20%)	
According to the procedure		
EUS‐CDS	19% (95% CI 15–25%)	99–272 days
EUS‐HGS	13% (95% CI 9–18%)	62–216 days
According to the stent type		
Plastic stent	28% (95% CI 21–38%)	97–272 days
Covered SEMS	14% (95% CI 10–20%)	72–216 days

*CDS* choledochoduodenostomy, *CI* confidence interval, *EUS* endoscopic ultrasound, *HGS* hepaticogastrostomy, *SEMS* self‐expandable metallic stent

The stent occlusion rate (stent dysfunction or re‐intervention) is reportedly 15% (70/434 cases) 11, 18, 23, 24, 26, 34, 54, 55, 56, 57, 58, 59, 60, 61, 62, 63, 64, 65, 66, 67. There is no significant difference in the stent occlusion rate between CDS (19%) and HGS (13%). The causes of stent obstruction and dysfunction, unlike those of EBD, are principally non‐neoplastic, including stent dislocation or biliary sludge.

Although comparison of stent patency results among studies is difficult due to use of different terminology and assessment criteria, the stent patency after EUS‐BD is 3–6 months 11, 18, 23, 24, 26, 34, 54, 55, 56, 57, 58, 59, 60, 61, 62, 63, 64, 65, 66, 67. A Japanese study 34 reported a mean time to stent dysfunction of 96 days: 103 days for EUS‐CDS and 62 days for EUS‐HGS. There was no significant difference in mean stent patency between covered self‐expandable metallic stents (SEMSs, 72 days) and plastic stents (97 days). Park et al. 13 reported a mean stent patency of 133 days: 152 days for CDS and 132 days for HGS. However, EUS‐BD resulted in a longer patency than did EBD in cases with duodenal stenosis or an indwelling duodenal stent 26, with EUS‐HGS showing a longer patency than that of EUS‐CDS 68.

Hara et al. 23, 24 conducted two clinical studies of EUS‐CDS as the initial biliary drainage modality using a plastic stent and covered SEMS. Although these were not randomized controlled studies, they involved the same subjects and institution, enabling comparison of the long‐term outcomes of EUS‐CDS between plastic stents 23 and covered SEMSs 24. The occlusion rate of plastic stents was 53%, compared with 11% for covered SEMSs, suggesting that covered SEMSs are superior in terms of stent patency.

No study has evaluated EUS‐BD re‐intervention for stent occlusion or dislocation. When stent occlusion develops, stent replacement, stent‐in‐stent, and/or stent cleaning via EUS‐BD are performed in many cases 23, 24, 66. It is necessary to assess the safety and efficacy of EUS‐BD as the initial procedure, as well as for re‐intervention.

There is no standardized method for assessing the outcomes of EUS‐BD; this hampers comparison of results among studies. EUS‐BD includes various procedures; thus, standardized criteria for assessing the outcomes (safety and efficacy) are needed. In addition, large‐scale multicenter prospective studies of the short‐term and long‐term outcomes should be conducted to avoid publication bias. The short‐ and long‐term outcomes are detailed in this section. However, standardization of the procedure and development of novel devices will significantly change the outcomes, and thus periodic updating of the guidelines is required.

### **Q6. What are the adverse events of EUS‐BD?**

**Adverse events of EUS‐BD include bile leakage, stent misplacement, hemorrhage, perforation, and peritonitis.**

**Mean voting score (range): 7.8 (5–9)**

A pooled analysis of early adverse events among studies involving ≥20 cases yielded an incidence of early adverse events of 14.6% (100/755 cases) 12, 13, 14, 15, 16, 17, 18, 34, 48, 51, 67, 69, 70, 71, 72, 73, 74, 75, 76, 77. The summary below includes all adverse events reported in each study (Table 6).

**Table 6:** Adverse event of EUS‐BD

	Incidence
EUS‐CDS	13.9% (20/144)
Bile leakage	2.8%
Stent migration	2.8%
Bleeding	2.5%
Perforation	1.4%
Peritonitis	1.4%
EUS‐HGS	18.2% (45/247)
Bleeding	3.7%
Bile leakage	2.8%
Biloma	2.6%
Stent migration	1.6%
Stent inward migration (IHBD, peritoneal cavity)	1.2%
Liver hematoma	1.2%
Sepsis	1.2%
EUS‐RV	12.4% (45/364)
Acute pancreatitis	2.7%
Pneumoperitoneum	2.2%
Biliary peritonitis	2.2%
Abdominal pain	1.9%
Bile leakage	1.4%
Bleeding	0.5%

*CDS* choledochoduodenostomy, *EUS* endoscopic ultrasound, *HGS* hepaticogastrostomy, *IHBD* intrahepatic bile duct, *RV* rendezvous

The incidence of early adverse events of EUS‐CDS, including biliary leakage (2.8%), stent migration (2.8%), hemorrhage (2.5%), perforation (1.4%), and bile peritonitis (1.4%), was 13.9% (20/144 cases) 13, 16, 18, 34, 69. Other adverse events included acute cholangitis 16, biloma 16, pneumoperitoneum 34, bilio‐arterial fistula 18, sepsis 18, abdominal pain 59, duodenal perforation 61, hemobilia 23, acute cholecystitis 63, and double punctures of the duodenum 78. Mortalities were also reported 18.

The incidence of early adverse events of EUS‐HGS, including hemorrhage (3.7%), bile leakage (2.8%), biloma (2.6%), stent migration (1.6%), stent misplacement (intrahepatic, intraperitoneal) (1.2%), intrahepatic hematoma (1.2%) and sepsis (1.2%), was 18.2% (45/247 cases) 13, 16, 18, 34, 67, 69, 70, 71. Other adverse events included perforation 16, abscess 16, acute cholangitis 34, pneumoperitoneum 18, intrahepatic hematoma 18, peritonitis 70, pseudoaneurysm of the hepatic artery 79, and biliary vomiting 80. Mortalities (2.0%) and cases of transfer to laparotomy (0.8%) were also reported 18.

The incidence of early adverse events of EUS‐RV, including acute pancreatitis (2.7%), pneumoperitoneum (2.2%), bile peritonitis (1.4%), and hemorrhage (0.5%), is 12.4% (45/364 cases) 12, 14, 15, 17, 48, 51, 72, 73, 74, 75, 76, 77. Other adverse events include perforation, hemorrhage (3.7%), bile leakage (2.8%), biloma (2.6%), stent migration (1.6%), stent misplacement (intrahepatic, intraperitoneal) (1.2%), intrahepatic hematoma (1.2%), and sepsis (1.2%). Perforation, leakage of contrast medium around the hepatoduodenal ligament, acute cholangitis, heart failure, respiratory failure, sepsis, and aspiration pneumonia have also occurred. Mortalities were also reported 14.

There were nine mortalities among a case report 81 and a series of ≥20 cases 14, 18, 37. The causes of morality were, for EUS‐CDS, bile peritonitis and sepsis in three cases each 18, for EUS‐HGS, sepsis due to stent dislocation and biliary peritonitis due to guidewire perforation 37, 81, and for EUS‐RV, one sudden death 4 days after the procedure 14.

These results should be interpreted with caution since most procedures were performed by experts and there might be overlapping cases, which might cause some biases.

### **CQ1. How can we select the EUS‐BD procedure?**

**EUS‐BD procedures are selected according to the patient's clinical condition, location of the bile duct obstruction, presence of duodenal obstruction, and gastrointestinal and biliary reconstruction.**

**[Recommendation level: 2, Evidence level D]**

**Mean voting score (range): 8.4 (7–9)**

**Figure 3:**
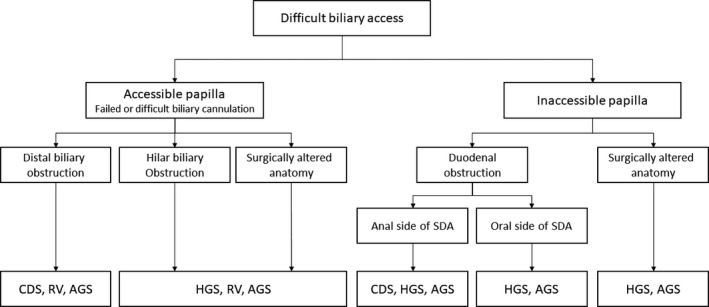
Flowchart of selection of various EUS‐BD procedures. *EUS* endoscopic ultrasound, *EUS‐AGS* EUS‐guided antegrade stenting, *EUS‐CDS* EUS‐guided choledochoduodenostomy, *EUS‐HGS* EUS‐guided hepaticogastrostomy, *EUS‐RV* EUS‐guided rendezvous technique, *SDA* supraduodenal angle

EUS‐BD includes EUS‐CDS, EUS‐HGS, EUS‐AGS, and EUS‐RV; the appropriate procedure should be selected based on their characteristics, location of the bile duct obstruction, presence of duodenal obstruction, gastrointestinal and biliary reconstruction, and the condition of the patient. EUS‐CDS is not indicated for hilar biliary obstruction, and thus EUS‐HGS, EUS‐AGS, or EUS‐RV should be used. If a transpapillary approach is difficult due to duodenal obstruction or ampullary invasion, EUS‐RV is not indicated. A retrospective study by Ogura et al. 68 suggested that EUS‐HGS showed better stent patency (HR 0.391, *P* = 0.045) in cases with duodenal obstruction, while EUS‐CDS was associated with adverse events, in particular reflux cholangitis (OR 10.285, *P* = 0.012). Figure 3 shows the suitability of the various EUS‐BD procedures according to the condition of the patient. In addition, EUS‐guided gallbladder drainage can be performed if bile duct puncture is unsuccessful, and it can achieve biliary drainage through the cystic duct. However, EUS‐guided gallbladder drainage can be performed only in cases with the patent cystic duct, which should be confirmed on EUS prior to the procedure 82. In contrast, in cases in which several approaches are possible, no comparative study has been reported, warranting further research.

Park et al. reported an algorithm for selection of the EUS‐BD procedure. According to this algorithm, EUS‐RV is the first choice for cases with failed ERCP but with an accessible ampulla, whereas EUS‐HGS or CDS is the first choice for cases with duodenal obstruction depending on the location of the biliary obstruction. Use of the algorithm yielded a success rate of >90% 51. Poincloux et al. based procedure selection on the clinical conditions, resulting in a similar safety profile and success rate to those reported by Park et al. 18. According to the algorithm developed by Khashab et al., EUS‐RV should be used for malignant biliary obstruction after failed ERC, and if EUS‐RV fails, EUS‐CDS/HGS is to be performed. The two procedures had similar effectiveness and safety profiles 76. Weilert 83 developed an algorithm, which selected EUS‐AGS using a transgastric intrahepatic bile duct approach for patients with failed ERCP or, if EUS‐AGS fails, then EUS‐HGS. EUS‐CDS was performed if EUS‐HGS failed or proved difficult. The intrahepatic bile duct approach was effective in 80% of cases. Iwashita et al. 50 reported the efficacy and safety of EUS‐AGS in patients with a surgically altered anatomy. Sirpum et al. 30 performed a systematic review of EUS‐BD in patients with a surgically altered anatomy and found that EUS‐BD after failed ERCP was as safe and effective as in those with a normal anatomy. However, the access route of EUS‐BD in patients with a surgically altered anatomy is often limited to the left intrahepatic bile duct, and EUS‐HGS may not be feasible in patients with surgical reconstruction. In addition, EUS‐RV requires access to the papilla, which can be difficult. Based on these findings, EUS‐CDS/HGS, EUS‐AGS, or EUS‐RV can be used for patients with a regular anatomy without duodenal obstruction; EUS‐CDS/HGS or EUS‐AGS can be performed in those with duodenal obstruction. The potential adverse events and patient condition described in Q6 must be considered when selecting a procedure.

Selection of EUS‐CDS or EUS‐HGS depends on the location of the bile duct obstruction. Prachayakul  and Aswakul 84 reported that HGS is applied for hilar biliary obstruction and CDS for distal biliary obstruction. The technical success, clinical success, and adverse event rates were 95.5%, 90.5%, and 9.5%, respectively. Gupta et al. 85 found no difference in the success and adverse event rates between EUS‐CDS and EUS‐HGS. In contrast, whereas both EUS‐CDS and EUS‐HGS can be used in patients with combined duodenal and distal biliary obstruction, Dhir et al. 86 showed in a retrospective study that EUS‐HGS has a significant risk of adverse events. Therefore, EUS‐CDS may be the first choice for patients with combined duodenal and distal biliary obstruction if the duodenal bulb is intact.

The patient's condition should be considered when selecting an EUS‐BD procedure. The location of biliary obstruction, presence of duodenal stricture, and reconstruction method in cases with a surgically altered anatomy should be evaluated by contrast enhanced computed tomography (CT), magnetic resonance imaging (MRI)/magnetic resonance cholangiopancreatography (MRCP), upper endoscopy, and surgical records. In addition, based on the low adverse event rate, if either EUS‐HGS or EUS‐CDS can be used, EUS‐CDS should be the first choice. Because both procedures are performed endoscopically, patient preference is not an issue. Moreover, the procedure is covered by public health insurance in Japan; thus, payment/reimbursement is not a consideration when selecting the EUS‐BD procedure.

### **CQ2. Which endoscope is recommended for EUS‐BD?**

**Only convex‐type EUS scopes can be used for EUS‐BD.**

**[Recommendation level 1, evidence level C]**

**Mean voting score (range): 8.4 (7–9)**

Several studies have used a convex (including linear)‐type EUS scope. A radial‐type EUS scope cannot visualize the needle route and thus is not suitable for EUS‐BD or EUS‐FNA. No prior study has directly compared EUS scopes. A forward‐viewing convex‐type EUS scope is reportedly useful for EUS‐CDS 24, 87. No other types of EUS scope can be used, and so patient preference is not a consideration.

### **CQ3. Which needle is recommended for EUS‐BD?**

**A 19 G FNA needle is recommended.**

**[Recommendation level 1, evidence level B]**

**Mean voting score (range): 8.7 (7–9)**

Following needle puncture, the procedure is conducted using a guidewire that passes through the puncture needle. Therefore, use of a 19‐G FNA needle is recommended and has been used in many previous studies. A 22‐G FNA needle may facilitate puncture of a smaller bile duct. However, a smaller guidewire (0.021 or 0.018 inches) does not allow device insertion, resulting in a lower success rate. Thus, a thin guidewire should be exchanged for a 0.025‐ or 0.035‐inch guidewire to complete the procedure. Whereas use of a needle knife is effective in cases with difficult puncture or mechanical dilatation 13, 23, 24, 85, the adverse event rate is higher 13, and thus routine use of a needle knife is not recommended. In Japan, there is no commercially available access needle for EUS‐BD, but a study using the needle knife has been performed outside of Japan 88. The needle knife has a needle‐shape stylet, and its tubular blunt outer sheath protects and facilitates manipulation of the guidewire. However, the needle knife has less ability for puncture and is not yet used globally.

In summary, a 19‐G EUS‐FNA needle is currently recommended for EUS‐BD. A puncture needle specifically designed for EUS‐BD is not commercially available, and guidewire insertion through the EUS‐FNA needle is not officially recommended. Therefore, development of needles for EUS‐BD is required. The patient is unaware of the puncture needle used for EUS‐BD but would prefer it to be effective and safe.

### **CQ4. Which guidewire is recommended for EUS‐BD?**

**A 0.035‐inch or hard‐type 0.025‐inch guidewire is recommended for ERCP.**

**[Recommendation level 2, evidence level C]**

**Mean voting score (range): 8.4 (7–9)**

Because no previous study has compared guidewires for EUS‐BD, we evaluated those used in previous reports. Whereas a 0.035‐inch guidewire was mainly used in the past, a stiff 0.025‐inch guidewire is now increasingly utilized for EUS‐BD, particularly in Japan. A short hydrophilic guidewire is reportedly useful for EUS‐RV 74.

If a 22‐G FNA needle is selected, a 0.021‐ or 0.018‐inch (small diameter) guidewire is used. However, guidewire manipulation is difficult and does not enable device insertion, hampering EUS‐BD. Therefore, a small diameter guidewire should be exchanged for a 0.025‐ or 0.035‐inch guidewire, which results in additional cost. There is no patient preference for guidewires, and so the guidewire should be selected based on its advantages and disadvantages. Coated guidewires are currently used for ERCP; these have a risk of shearing and fracture when passed through a EUS‐FNA needle. No guidewire has been approved for EUS‐BD, and so a guidewire with a good safety profile and high maneuverability is needed.

### **CQ5. Which dilatation device is recommended for EUS‐BD?**

**A bougie dilator and balloon dilator are recommended, and a co‐axial electric cautery dilator can be used.**

**[Recommendation level 2, evidence level C]**

**Mean voting score (range): 8.2 (6–9)**

Dilatation of a fistula is an important but difficult step during EUS‐BD and facilitates insertion of various devices including stents. EUS‐BD can be unsuccessful due to failure of this step. Mechanical dilatation using a biliary dilatation catheter (bougie catheter) or balloon dilator or cautery dilation using a co‐axial cautery dilator is performed 71, 89, 90, 91, 92, 93. Although dilatation of a fistula using an electric cautery device is technically easy, it has risks of bleeding, perforation, penetration, and incorrect puncture. Therefore, an electric cautery device should be used if a non‐cautery device is not effective. Use of a non‐coaxial needle knife is associated with an increased adverse event rate and thus not recommended 13, 94. Overdilation of the fistula may increase the risk of bile leak and the type and size of dilators should be selected according to the stent. No device has been approved by the regulatory authority, and thus development of such a device is needed. Patients do not have a preference for a dilatation device; thus, the device can be selected based on its efficacy and safety.

### **CQ6. Which stent is recommended for EUS‐BD?**

**A covered SEMS is recommended. A plastic stent can be considered based on the condition of the patient.**

**[Recommendation level 2, evidence level B]**

**Mean voting score (range): 7.9 (5–9)**

Early after its development, the outcomes of plastic stents with EUS‐BD were reported 23, 95. However, these stents are associated with risks of bile leakage, fistula, and stent migration 86. Therefore, covered SEMSs have been used recently 24, 57, 60, 62, 64, 85, 94, 96, 97, 98. In a retrospective analysis of 121 EUS‐BD 94, the use of a plastic stent was significantly associated with adverse event rate (OR 4.95, *P* = 0.01). Although a dedicated plastic stent for EUS‐BD has been developed, the rate of adverse events was 17.4% (three mild pain and one moderate bleeding) in a prospective feasibility study 67, which appeared comparable to the conventional plastic stents. Use of braided‐type SEMSs with a high shortening rate requires some technical skill because of the potential for adverse events, such as stent migration or dislocation 81, 99. Therefore, >10 cm SEMSs are recommended when SEMSs without anti‐migration systems are used 100. To reduce the risk of side branch obstruction or migration, a partially covered SEMS that is uncovered at the haptic end is used. However, because such stents are prone to migration at the gastric end, ≥10 cm stents are currently used 101. Plastic stents reduce the frequency of adverse events, such as segmental cholangitis or liver abscess caused by peripheral bile duct obstruction 67. Studies of a biflanged lumen‐apposing metal stent (AXIOS®, Boston Scientific, Marlborough, MA, USA) for EUS‐CDS 102, 103, 104, 105 and of other types of dedicated stents for EUS‐CDS or EUS‐HGS have been performed in Korea 65, 91, 106. In a prospective multicenter study of a lumen‐apposing metal stent with cautery‐enhanced delivery system 107, EUS‐CDS was technically successful in 100% and jaundice resolved in 95%. Of note, there were no procedure‐related adverse events including bile leak. Meanwhile, a sump syndrome similar to surgical choledochoduodenostomy was reported after EUS‐CDS using a lumen‐apposing metal stent 108. A comparative study of a lumen apposing metal stent and a conventional stent is warranted. Currently, 8‐to‐10‐mm‐diameter and 4‐to‐6‐cm‐length SEMSs are used for EUS‐CDS and 8‐to‐10‐mm‐diameter and 10‐to‐12‐cm‐length SEMSs for EUS‐HGS. Patients appear to have no preference regarding stents, and so covered SEMSs are recommended based on their efficacy and safety. However, in patients in whom covered SEMSs are difficult to use, a plastic stent can be applied, and therefore stent selection should be based on the clinical condition of the patient. No stent has been approved specifically for EUS‐BD by the Japanese regulatory authority, and the development of such a stent is needed.

### **CQ7. What supportive treatment is recommended for EUS‐BD?**

**Antibiotic treatment is suggested for patients with concomitant cholangitis.**

**[Recommendation level 2, evidence level D]**

**Mean voting score (range): 8.3 (7–9)**

The purpose of supportive therapy is to prevent adverse events. However, no study has assessed the effectiveness of prophylactic treatment for EUS‐BD, and thus no established supportive therapy exists. In the Antibiotic Prophylaxis for GI Endoscopy guidelines of the American Society of Gastrointestinal Endoscopy 109, the use of antibiotics is recommended in patients with acute cholangitis at the time of trans‐papillary endoscopic biliary drainage, as well as in those with anticipated incomplete biliary drainage. In the Antibiotic Prophylaxis for GI Endoscopy guidelines of the British Society of Gastroenterology 110, antibiotic prophylaxis for prevention of sepsis or cholangitis is recommended for patients with concomitant cholangitis/sepsis at the time of ERCP, or for those with anticipated difficult or failed complete biliary drainage. Based on these guidelines, antibiotic prophylaxis should be performed in patients with concomitant cholangitis at the time of EUS‐BD. In the real‐world setting, however, antibiotic prophylaxis is performed in most centers, even for patients without concomitant cholangitis, to prevent potential peritonitis or progression of peritonitis due to leakage of bile or gastrointestinal contents. As there is little evidence of the necessity of antibiotic prophylaxis for EUS‐BD, further studies are necessary. Antibiotics have potential side‐effects and may cause development of antibiotic‐resistant bacteria. Therefore, patients may have various preferences for antibiotic prophylaxis.

### **CQ8. What is recommended for the management procedures after EUS‐BD?**

**Careful observation of patients is required after EUS‐BD, and adequate treatment should be carried out when adverse events are suspected.**

**[Recommendation level 2, evidence level D]**

**Mean voting score (range): 8.1 (5–9)**

There are no previous reports on the management or observation methods conducted after EUS‐BD. Post‐procedure management is required for early detection and diagnosis of adverse events. Monitoring of symptoms and vital signs (blood pressure, pulse rate, body temperature) is required, and when symptoms (fever, abdominal pain, vomiting, hematemesis, and melena) or vital signs suggest adverse events, physical examination, blood tests, and contrast‐enhanced CT should be performed immediately. Abdominal plain CT the day after EUS‐BD is useful for assessing stent position, biliary drainage status, and adverse events (e.g. perforation, bile leakage, intra‐abdominal emphysema) and is performed in many Japanese institutions. When routine CT is not available, at least a plain radiograph of the abdomen should be performed on the next day of the EUS‐BD procedure. When clinical signs and symptoms as well as the plain radiograph suggest the possibility of adverse events such as stent dislocation or perforation, then CT should be considered. When adverse events are suspected, the surgeon and radiologist should be consulted regarding the appropriate treatment. Adverse events such as severe infection, sepsis, and shock can be lethal if overlooked or left untreated. Efforts to prevent such severe adverse events are thus vital.

### **CQ9. What is recommended for the prevention and treatment of adverse events of EUS‐BD?**

**There are no established prevention or treatment protocols for adverse events of EUS‐BD. The indications for EUS‐BD should be determined based on the potential adverse events by experienced endosonographers with the skill and experience to address them.**

**[Recommendation level 2, evidence level D]**

**Mean voting score (range): 7.4 (5–9)**

Because prevention and treatment protocols for adverse events of EUS‐BD have not been established, the procedure should be carried out by experienced endosonographers who can handle the adverse events. Inexperienced endosonographers may not complete the procedure or may misplace the stent, which can lead to severe adverse events. Furthermore, inadequate management of adverse events can lead to mortality. Safety is important from the patients’ point of view; the recommendations are acceptable to patients.

Although there are no established prevention or treatment protocols, the following are recommended for adverse events of EUS‐BD. EUS‐BD can result in two types of adverse events: those related to the procedure and those following successful EUS‐BD. Thus, EUS‐BD should be performed carefully, and patients should be observed post‐procedure to enable timely identification and treatment of adverse events.

#### Adverse events related to the procedure

Although preventing bile leakage and pneumoperitoneum is difficult, avoiding excess fistula dilation and minimizing the delay between fistula dilatation and stent placement reduce the risk of bile leakage and bile peritonitis. A single step device of lumen‐apposing metal stents with cautery‐enhanced system can potentially reduce the risk of bile leak during EUS‐CDS procedure. To prevent retained pneumoperitoneum, CO_2_ insufflation should also be used whenever possible. In addition, the use of covered SEMSs is recommended to prevent bile leakage. Bile leakage can also occur in cases with plastic stents if the fistula is larger than the stent diameter. Therefore, the fistula and stent should be of similar size. Stent release inside the scope channel can prevent intraperitoneal stent deployment 111, 112. After stent misplacement, an additional stent can be placed if the guidewire is kept in place. Thus, the guidewire should not be removed until appropriate stent placement is confirmed 113.

To avoid bleeding during EUS‐BD, blood vessels along the puncture route, including collateral ones, should be evaluated by contrast CT or color doppler EUS imaging. The EUS scope should not be allowed to compress the surrounding vessels, as this hampers their recognition by doppler EUS imaging. In addition, the use of a needle knife, which is not coaxial to the indwelling guidewire, should be avoided. To prevent bleeding from the fistula, the use of a covered SEMS is recommended. Stent placement after double puncture of the duodenum during EUS‐CDS can cause perforation. Therefore, careful observation of the duodenal wall by EUS, endoscopic visualization of the guidewire location, or another method of confirmation should be performed. Dilatation of the duodenum with water prior to needle puncture allows visualization of the folded duodenal mucosa by EUS 114. In addition, direct visualization using a forward‐viewing convex echoendoscope can prevent double puncture.

To avoid esophageal puncture, which can cause hemothorax, pneumothorax, mediastinitis, or mediastinal emphysema, clipping at the esophagogastric junction enables confirmation of the position of the esophagus under the fluoroscope. If the esophagus is punctured during EUS‐RV, the fistula should not be dilated using a dilator. Also, if the esophagus is punctured during EUS‐HGS, the procedure should be terminated. Endoscopic observation of the puncture site can prevent severe adverse events during fistula dilatation or stent placement.

##### Management of adverse events

If severe adverse events such as peritonitis, bile leakage, or pneumoperitoneum are suspected, CT, preferably contrast‐enhanced CT, should be performed to determine the appropriate timing of surgery. Procedure‐related abdominal pain due to excessive insufflation, needle puncture, fistula dilation, or stent placement should improve within a short time.

Acute cholangitis is caused by failed stent placement or by stent misplacement, dislocation, or obstruction. Adequate drainage to manage acute cholangitis should be ensured after CT examination. If drainage is successful and temporary cholangitis confirmed, antibiotic treatment is effective. If cholangitis in a non‐drainage area (segmental cholangitis) is suspected, conservative treatment with antibiotics is initiated, and additional drainage should be performed is there is no clinical response. The risk of segmental cholangitis should be considered when placing covered SEMSs.

If bleeding is observed at the puncture site, endoscopic hemostasis should be performed. In cases of fistula bleeding, vascular injury should be considered, which can be caused by puncture, dilation, a needle knife, and/or an electric cautery dilator. Embolization using IVR is needed if a pseudoaneurysm is suspected on contrast‐enhanced CT. Otherwise, replacement with a larger stent or covered SEMS can accomplish hemostasis in most cases.

Pleural drainage or surgical interventions are required in some cases of hemothorax, mediastinitis, or symptomatic mediastinal emphysema.

### **CQ10. What is necessary for EUS‐BD credentials?**

**EUS‐BD should be performed by an EUS‐BD expert with sufficient clinical experience in both ERCP and EUS‐FNA or by expert endoscopists under the supervision of an EUS‐BD expert.**

**[Recommendation level 2, evidence level D]**

**Mean voting score (range): 8.4 (7–9)**

During EUS‐BD, many devices for ERCP are used, and the puncturing and endoscope manipulation are similar to those used for EUS‐FNA. Therefore, EUS‐BD should be performed by expert endoscopists with sufficient clinical experience in both ERCP and EUS‐FNA. Unexperienced endoscopists alone may be unable to complete the procedure or may place the stent in the wrong location, possibly leading to fatal adverse events. The endoscopist should be able to perform EUS‐BD safely and effectively.

It is recommended that endoscopists be trained directly by an experienced EUS‐BD expert; procedure‐training videos and self‐training are not sufficient. Observation of or assistance with the procedure is the first step in learning EUS‐BD, and trainees should begin with a simple EUS‐BD procedure under the supervision of an EUS‐BD expert. When a complicated EUS‐BD is performed by trainees, an EUS‐BD expert should supervise and replace the trainee if they cannot complete the procedure. Thus, EUS‐BD should be introduced after a short‐term training program in institutions with experienced EUS‐BD experts. Having an EUS‐BD expert supervise the procedure is also recommended.

The American Society of Gastrointestinal Endoscopy recommends that endosonographers complete a minimum of 150 supervised EUS procedures, consisting of 75 pancreaticobiliary and 50 EUS‐FNA (25 submucosal lesions and 25 pancreatic lesions) procedures, to achieve competence in all aspects of EUS 115. The European Society of Gastrointestinal Endoscopy recommends that endosonographers learn to handle a convex EUS scope to visualize the lesion by him/herself prior to learning EUS‐FNA and subsequently undergo training for EUS‐FNA (involving 30 pancreatic and 20 non‐pancreatic lesions) under the supervision of an EUS‐BD expert 116. Oh et al. 117 reported that the procedure time and the adverse event rate of EUS‐HGS stabilized after 33 cases but a learning curve for EUS‐BD should be further investigated.

### **CQ11. What is recommended for EUS‐BD training?**

**Endosonographers should be trained by experts at experienced institutions. The training consists of lectures followed by demonstration of the procedure and hands‐on‐training.**

**[Recommendation level 2, evidence level D]**

**Mean voting score (range): 8.4 (5–9)**

The recommended training steps are described below.

The technical success and adverse event rates of EUS‐BD, and the management of adverse events, differ between physicians who have and those who have not undergone the recommended training steps. Patients want to receive EUS‐BD by a physician with sufficient training. However, there is no official training program in Japan.

Step 1: An endoscopy expert with sufficient clinical experience teaches the procedure using the standard visualization of convex EUS 118 or videos. Recently, training simulators and models for EUS‐BD have been developed 119, 120; these are useful for acquiring basic knowledge of the procedure.
Step 2: By visiting an expert institution and participating in live demonstrations of the procedure, the trainees receive instruction on the requirements for EUS‐BD (e.g. the preparation, process, and roles of medical staff).
Step 3: An EUS‐BD expert with sufficient clinical experience with EUS‐BD and sufficient training experience provides hands‐on clinical training. First, the trainer demonstrates the procedure. Next, the trainee participates in the procedure as an assistant to fully understand each step and device manipulation. The trainee subsequently performs the procedure by her/himself beginning with less complicated procedures under the direct supervision of the trainer, who will handle any difficult situations. Supervision by the trainer should continue until the trainee can perform the procedures by her/himself without difficulty.

### **CQ12. What is recommended for the institutional requirements for EUS‐BD?**

**EUS‐BD should be performed in institutions where support from surgeons and radiologists is available.**

**[Recommendation level 2, evidence level D]**

**Mean voting score (range): 8.1 (5–9)**

Each institution should establish a multidisciplinary team comprising surgeons, radiologists, IVR experts, nurses, and medical administrators to support EUS‐BD. An EUS‐BD database should be constructed to enable regular evaluation of the outcomes and the adverse event rate in comparison with other expert centers. If the EUS‐BD outcomes are unsatisfactory, or the adverse event rate is high, the underlying reasons should be explored. In addition, consultation with experienced endoscopists should be considered 121.

### **CQ13. What are the ethical considerations for EUS‐BD?**

**It is recommended that EUS‐BD be performed carefully after considering the indications and obtaining informed consent from the patient.**

**[Recommendation level 2, evidence level D]**

**Mean voting score (range): 8.4 (7–9)**

EUS‐BD was approved and covered by the Japanese public health insurance system in 2012, but evidence of its safety and long‐term outcomes is lacking. Given the current status of EUS‐BD, the indications should be determined based on four medical ethics principles 122: “autonomy”, “non‐maleficence”, “beneficence”, and “justice.” The indications for EUS‐BD should be discussed with, and the treatment should be selected by, the patient after providing fully informed consent 123. To this end, it is critical to inform the patient of the procedure safety and effectiveness and of other treatment options. Other treatment options such as PTBD, ERCP, and surgery must be fully explained to enable understanding of the advantages and disadvantages of EUS‐BD.

Institutional review board approval for EUS‐BD should be obtained for cases with indications without clinical evidence, those outside the standard treatment protocol, or if a new device is to be used. It is mandatory to inform the patient that she/he can withdraw their consent at any time without unfavorable consequences. The explanations provided to the patient, as well as their consent, must be documented in medical records. Furthermore, EUS‐BD must be performed by expert endosonographers with sufficient knowledge and experience to handle adverse events or by skilled endoscopists under the supervision thereof.

## Conflict of interest

Research grant: Boston Scientific Japan K.K., Century Medical, Inc., Gadelius Medical K.K., Piolax Medical Devices, Inc., FUJIFILM Medical Co., Ltd. Lecture fee: Boston Scientific Japan K.K., Gadelius Medical K.K., Medico's Hirata Inc., Olympus Corporation.

## Appendix 2

The members of the guidelines committee who created and evaluated these guidelines are listed below.

### GUIDELINE CREATION COMMITTEE

Chair: Hiroyuki Isayama (Department of Gastroenterology, Graduate School of Medicine, Juntendo University, Tokyo, Japan).

Vice‐Chair: Takao Itoi (Department of Gastroenterology and Hepatology, Tokyo Medical University, Tokyo, Japan).

Ichiro Yasuda (Third Department of Internal Medicine, University of Toyama, Toyama, Japan), Masayuki Kitano (Second Department of Internal Medicine, Wakayama Medical University, Wakayama, Japan), Atsushi Irisawa (Department of Gastroenterology, Dokkyo Medical University, Tochigi, Japan), Shomei Ryozawa (Department of Gastroenterology, Saitama Medical University International Medical Center, Saitama, Japan), Akio Katanuma (Center for Gastroenterology, Teine‐Keijinkai Hospital, Sapporo, Japan), Hiroshi Kawakami (Division of Gastroenterology and Hepatology, Department of Internal medicine, Faculty of Medicine, University of Miyazaki, Miyazaki, Japan), Yousuke Nakai (Department of Gastroenterology, Graduate School of Medicine, The University of Tokyo, Tokyo, Japan), Kazuo Hara (Department of Gastroenterology, Aichi Cancer Center, Nagoya, Japan), Takuji Iwashita (First Department of Internal Medicine, Gifu University Hospital, Gifu, Japan).

### EXPERT PANELIST COMMITTEE

Chair: Naotaka Fujita (Miyagi Health Check‐up Plaza, Sendai, Japan).

Kenji Yamao (Department of Gastroenterology, Narita Memorial Hospital, Nagoya, Japan), Hiroyuki Isayama (Department of Gastroenterology, Graduate School of Medicine, Juntendo University, Tokyo, Japan), Takao Itoi (Department of Gastroenterology and Hepatology, Tokyo Medical University, Tokyo, Japan), Ichiro Yasuda (Third Department of Internal Medicine, University of Toyama, Toyama, Japan), Masayuki Kitano (Second Department of Internal Medicine, Wakayama Medical University, Wakayama, Japan), Atsushi Irisawa (Department of Gastroenterology, Dokkyo Medical University, Tochigi, Japan), Shomei Ryozawa (Department of Gastroenterology, Saitama Medical University International Medical Center, Saitama, Japan), Akio Katanuma (Center for Gastroenterology, Teine‐Keijinkai Hospital, Sapporo, Japan), Hiroshi Kawakami (Division of Gastroenterology and Hepatology, Department of Internal medicine, Faculty of Medicine, University of Miyazaki, Miyazaki, Japan), Takuji Iwashita (First Department of Internal Medicine, Gifu University Hospital, Gifu Japan).

### EVALUATION COMMITTEE

Chair: Kazuo Inui (Department of Gastroenterology, Fujita Health University Bantane Hospital, Nagoya, Japan).

Masanori Sugiyama (Department of Surgery, Tokyo Rosai Hospital, Tokyo, Japan), Kenjiro Yasuda (Department of Gastroenterology, Kyoto Second Red Cross Hospital, Kyoto, Japan), Keiichi Kubota (Second Department of Surgery, Dokkyo Medical University, Tochigi, Japan).

### EXTERNAL EVALUATION COMMITTEE

Chair: Tadahiro Takada (Department of Surgery, Teikyo University School of Medicine, Tokyo, Japan).

Masahiro Yoshida (Department of Hepato‐Biliary‐Pancreatic and Gastrointestinal Surgery, International University of Health and Welfare, Ichikawa Hospital, Ichikawa, Japan).
